# Towards East Asian Facial Expression Recognition in the Real World: A New Database and Deep Recognition Baseline

**DOI:** 10.3390/s22218089

**Published:** 2022-10-22

**Authors:** Shanshan Li, Liang Guo, Jianya Liu

**Affiliations:** 1 School of Mathematics and Statistics, Shandong University, Weihai 264209, China; 2 Data Science Institute, Shandong University, Jinan 250100, China

**Keywords:** facial expression recognition, East Asian, real-world database, neural network

## Abstract

In recent years, the focus of facial expression recognition (FER) has gradually shifted from laboratory settings to challenging natural scenes. This requires a great deal of real-world facial expression data. However, most existing real-world databases are based on European-American cultures, and only one is for Asian cultures. This is mainly because the data on European-American expressions are more readily accessed and publicly available online. Owing to the diversity of huge data, FER in European-American cultures has recently developed rapidly. In contrast, the development of FER in Asian cultures is limited by the data. To narrow this gap, we construct a challenging real-world East Asian facial expression (EAFE) database, which contains 10,000 images collected from 113 Chinese, Japanese, and Korean movies and five search engines. We apply three neural network baselines including VGG-16, ResNet-50, and Inception-V3 to classify the images in EAFE. Then, we conduct two sets of experiments to find the optimal learning rate schedule and loss function. Finally, by training with the cosine learning rate schedule and island loss, ResNet-50 can achieve the best accuracy of 80.53% on the testing set, proving that the database is challenging. In addition, we used the Microsoft Cognitive Face API to extract facial attributes in EAFE, so that the database can also be used for facial recognition and attribute analysis. The release of the EAFE can encourage more research on Asian FER in natural scenes and can also promote the development of FER in cross-cultural domains.

## 1. Introduction

In recent years, facial recognition technology has become increasingly mature. Facial expression recognition (FER), as an important branch of facial recognition, has also received growing attention from researchers. At present, It is widely used in human–computer interaction, animation, medical care, public safety, and other fields [1]. Over the past decades, FER research has been conducted using datasets obtained by posing in controlled laboratory conditions. As the research has progressed, it has been found that the algorithms obtained by training on these datasets will have large deviations in practical applications. This is mainly because there are various head poses, illuminations, and occlusions in the real world. However, the lab-controlled datasets have consistent conditions, which are not enough to cope with various changes in the complex real world. Some researchers have compared the performance of existing FER algorithms on commonly used datasets. The experimental results showed that these algorithms had a better overall performance on lab-controlled datasets. However, for datasets in the real scene, the recognition accuracy was significantly reduced [2].

To solve this problem, the focus of FER has recently shifted to challenging natural scenes. In addition, the open-source nature of some large real-world datasets such as AffectNet [3], EmotioNet [4], RAF-DB [5], and AFE [6] has promoted the application of FER technology in the real world. However, most of these datasets are based on European-American cultures, and only a few of them are based on Asian cultures. This is because the data for the former are more readily and publicly available online. It has been demonstrated that emotional expression is not universal but culturally different [7]. Chen et al. [6] used the European-American culture-based RAF-DB and the Asian culture-based AFE as source data, respectively, to test the recognition results of the same algorithm on target data from several other different cultures. Experimental results showed that using AFE as source data outperformed the results of using RAF-DB as source data in recognizing Asian culture-based datasets and vice versa, proving the cultural differences in emotional expression. In addition, some external factors, such as cultural background, environment, and personal experience, can make different cultural groups differ in identifying the same facial expressions [8,9], resulting in inconsistent labeling criteria of existing datasets. Due to these differences, the existing European-American cultural datasets are not suitable for the study of Asian FER.

Owing to the diversity of huge data, FER in European-American cultures has recently developed rapidly. In contrast, the development of FER in Asian cultures is limited by the data. To facilitate the development of Asian FER in the real world, we create a challenging real-world East Asian facial expression database that covers various head pose transformations, illumination changes, and personal attributes. All data are collected from movies that are closest to real scenes, then labeled into seven expressions: angry, disgust, fear, happy, neutral, sad, and surprise. Furthermore, we use the Microsoft Cognitive Face API to extract facial attributes, including age, gender, head rotation angle, and occlusion. All these attributes are saved in annotation files so that future users can select appropriate data to meet their needs.

The database can be used for action recognition in the sensing environment, especially for facial expression recognition. The camera in the action recognition device captures the facial images; then, the integrated image algorithm chip analyzes them. The recognition results can be applied in many aspects. For example, in game production, the camera in front of the screen can be used to obtain the player’s emotional changes and make real-time feedback accordingly to enhance the immersive experience; in animation production, the changes of facial expressions of actors can be captured by the camera to generate the movement and expression of the virtual character; in medical treatment, the camera can be integrated into wearable devices to observe the mood fluctuations of autistic patients in real-time to assist in rehabilitation treatment; in public safety, the use of surveillance cameras in various places can help determine whether pedestrians have abnormal emotions to prevent crime.

This paper is organized as the following. We first review previous studies and identify the gaps in the field of FER. Then, we describe how our database is constructed. Finally, we conduct a series of experiments to demonstrate the value of our database.

## 2. Related Work

### 2.1. Existing Databases

In this section, we will introduce the existing Asian facial expression databases, and detailed information on them is presented in Table 1, including collection environment, number of images, number of subjects, expression category, etc.

The Japanese Female Facial Expression (JAFFE) [10] database is a commonly used data set. It contains ten young Japanese women, each corresponding to three or four posed images for each expression (six basic expressions [11]: angry, disgust, fear, happy, sad, surprise, and neutral). Before photo-shooting, subjects were asked to have their faces uncovered by the hair to reveal all expression areas. Images were taken from the front with faces in the center and were finally saved in an uncompressed grayscale format. This data set captured seven expressions from each subject, allowing a comprehensive study to compare the variation between different expressions. However, it only contains expression data of young women and cannot be combined with gender and age for further analysis.

The Taiwanese Facial Expression Image Database (TFEID) [12] consists of 40 subjects with an equal ratio of male and female. All subjects were asked to perform eight expressions (six basic expressions, contempt, and neutral) at different intensities (high and slight). The expressions were simultaneously captured by two cameras placed at different angles (0° and 45°). Finally, 7200 image sequences were collected, but only a part of the data set is publicly available, including 268 images with six basic expressions and neutral. Due to the small number, this data set is poorly generalized and is only suitable for expression recognition in a single environment.

The POSTECH Face Database (PF07) [13] captured the expressions of 100 males and 100 females. The subjects were required to pose for four different expressions (happy, surprise, anger, neutral), which were simultaneously captured by five cameras placed in the front, upper, lower, left, and right of subjects at 22.5° intervals. In addition, each angle of each expression corresponds to 16 different illumination conditions, so each subject corresponds to 320 images. This data set takes into account the effects of angle and illumination and is more robust. The disadvantage of this database is that only four expressions were collected, making it impossible to compare them directly with other datasets.

The Natural Visible and Infrared Facial Expression Database (NVIE) [14] is the first lab-controlled facial expression database that contains both natural visible and infrared images. All subjects were college students, among whom 108 participated in posed expressions and 215 participated in spontaneous expressions induced by film clips. The effects of illumination direction (front, left, and right) and glasses (with and without glasses) were considered during the acquisition process, and images were taken simultaneously by a visible and infrared camera directly in front of the subjects. However, because spontaneous expressions were uncontrolled, ultimately only disgust, fear, and happy expressions were successfully induced. The database contains both visible and infrared expression data, so it can be used for multi-modal expression analysis.

The Chinese affective face picture system (CAFPS) [15] collected images from 220 college students, middle-aged and elderly people, and children. Subjects were asked to remove jewelry such as earrings and necklaces before photo acquisition. All images were taken directly in front of the subjects and saved as grayscale images. During post-processing, external features such as hair, ears, and neck were removed, and only facial features were kept. This data set belongs to the Chinese face standard system, which is widely used in localized emotion research.

The Kotani Thermal Facial Emotion (KTFE) database [16] contained both visible and thermal facial expressions. Twenty-six subjects from Vietnam, Thailand and Japan participated in the experiment. Seven different types of emotional video clips were used to induce subjects to generate corresponding emotions. All expressions were spontaneously generated and there were no uniform requirements for the subject’s head posture and whether to wear glasses. During the experiment, the light was kept uniform, and both visible and infrared images were collected by the infrared camera directly in front of the subjects. The database contains expression data of infrared light, so it can also be used for infrared and spectral expression analysis.

The Korea University Facial Expression Collection-Second Edition (KUFEC-II) [17] consists of 57 professional actors (28 males and 29 females) who were trained to pose for photographs. All subjects were dressed in uniform and were instructed not to wear makeup, not to have exaggerated hair color, and to have their hair pinned to reveal the hairline and ears. Images were taken simultaneously by three high-resolution cameras placed around the subjects (45° apart from the front and left). After photo shooting, the most representative 399 images were selected and then labeled in four dimensions (validity, arousal, type, and intensity). The data set was created strictly based on FACS, so the participants’ facial expressions and actions were very standard. It is very suitable for application in the field of animation production, which can assist in generating the movements and expressions of virtual characters.

The Taiwanese facial emotional expression stimuli (TFEES) data set [18] is a combination of the existing database TFEID and images acquired in their study. The stimulus from TFEID consisted of 1232 frontal view facial expression images of 29 Taiwanese actors, and the remaining 2477 images of 61 Taiwanese were taken during the study. All participants were asked to remove eyeglasses and perform expressions according to AUs described in the FACS. Images were captured by a camera placed directly in front of the participant. Although the database contains many expression images, they are not applicable to the recognition of real scenes because the expressions are posed.

The Kokoro research center facial expression database (KRC) [19] collected images from 74 college students (50 males and 24 females). All subjects performed expressions according to the FACS examples provided. In addition to looking straight ahead, subjects were asked to look from straight ahead to left and right, respectively, while keeping their faces still to capture expressions with averted gazes. Three cameras were used to take pictures of subjects in front, left, and right simultaneously, and the subjects’ eyes were uniformly positioned in the center of the pictures. The database is designed specifically for psychological experiments. In addition to the basic expression categories, it is labeled with rating intensity and discrimination performance.

The Tsinghua facial expression database (Tsinghua FED) [20] is a data set containing different age groups, including 63 young and 47 older adults. Subjects were asked to have their face free of tattoos and accessories such as glasses, necklaces, and earrings and to have no makeup to conceal the actual age or the texture of the skin. Eight expressions (six basic expressions, neutral and content (smile without teeth)) were induced by a three-stage emotion induction method. Images were taken directly in front of the subjects and saved in color in JPEG format. The data set provides facial expressions in two age groups, young and old, which can be used to study the changes in facial expressions of East Asians from young to old.

The Micro-and-macro Expression Warehouse (MMEW) [21] contains both macro and micro expressions. The subjects were 30 Chinese (23 males and 7 females), and all of them were induced to produce six basic expressions through different types of emotional videos. Then, the expressions were captured by a camera placed in front of them. The faces were eventually cropped from the images and saved with a resolution of 231 × 231. As no relevant requirement has been made to wear glasses or not, the database therefore contains some expressions with glasses. The data set contains both macro and micro expressions of the same subject, so it can be used to study the differences between them.

Although the databases presented above are publicly available, they were all captured under laboratory-controlled conditions. All participants were required to perform the given expressions or to induce the corresponding emotions through video clips. Such controlled expressions are not sufficient to cope with the complex changes of the real world. In recent years, people have gradually realized the importance of recognizing facial expressions in real scenes. This is a very complex and challenging problem that relies on the amount and diversity of facial expression data.

The Asian Face Expression (AFE) [6] database is the first large data set of Asian facial expressions in natural scene conditions. The raw images contain about 500,000 Asian face images downloaded from Douban Movie. Each image was annotated by three or four annotators based on seven expressions (six basic expressions and neutral), and only images where all annotations were the same were kept, resulting in 54,601 images. This amount is sufficient for training large models. Moreover, the expressions in movies are diverse and can cope with the changes of real life.

However, only one real-world Asian facial expression database, AFE, currently exists, which is far below the number of those of European-American cultures, both in terms of the number of databases and the amount of expression data in the databases. To promote the development of Asian FER in natural scenes, large amounts of expression data generated in real life are urgently needed.

### 2.2. Existing Algorithms

FER can be divided into three stages [22]: pre-processing, feature extraction, and feature classification, among which feature extraction is the most important, and the accuracy of classification depends largely on the effectiveness of the extracted features [23].

In the pre-processing stage, face detection is usually used to extract facial regions. The most commonly used algorithms are Viola-Jones [24] and MTCNN [25]. The former can stably detect faces in the data set under lab-controlled conditions, but its performance in complex environments decreases substantially. The latter uses a deep learning approach with higher robustness and superior performance in complex natural scenes [2,26]. Moreover, MTCNN can perform face alignment processing at the same time while ensuring real-time performance. The disadvantage is that MTCNN will miss detection when the number of faces in the image is relatively large. Overall, it is still a good choice for face detection. To reduce the influence of illumination and head pose, some studies are also working on pre-processing images using illumination normalization [27] and pose normalization [28]. Additionally, some methods of data enhancement are also widely used to increase data diversity for better training of classification models [29].

The main function of feature extraction is to extract the most representative and descriptive information from images. Traditional feature extraction algorithms are based on artificially designed features, which can be roughly divided into five categories. Among them, texture feature-based methods include Gabor [30], LBP [31] and its variants such as LDP [32] and WPLBP [33], edge feature-based methods including HOG [34] and its variants PHOG [30], global and local feature-based methods include PCA [35] and SWLDA [36], geometric feature-based methods including LCT [37], and salient patch-based methods [38]. These methods were dominant in the past, mainly because previous studies were based on data with controlled conditions. In recent years, deep learning has shown powerful feature extraction capabilities. It can acquire high-level abstract features through multiple layers of nonlinear transformations. Deep learning methods such as convolutional neural networks (CNN) [27], deep belief networks (DBN) [39],deep autoencoders (DAE) [40], and generative adversarial networks (GAN) [41] are gradually gaining popularity among researchers. CNN relies on a set of learnable filters to extract features and is robust to face position and scale changes. However, the network structure needs to be carefully designed, and the larger the model, the higher the training cost. DBN is a directed graph model, it can learn the deep hierarchical representation of training data, and the algorithm is very simple to sample. However, the non-decomposability of the probabilistic graph makes the posterior computation very difficult. Both DAE and GAN are usually used in data generation tasks, they employ unsupervised learning (for unlabeled data) for feature extraction and feature representation. DAE uses an encoder–decoder structure to reconstruct its input by minimizing the reconstruction error. However, this will result in local oversight of the data rather than global access to information. GAN is based on the idea of adversarial generation. The discriminator judges the data as a whole and has a better grasp of the global information. However, the random input of the generator has a weak correspondence with the data, which leads to poor control of the generation quality. In general, CNN is widely used in FER due to its unique pattern recognition advantages.

Feature classification is the last stage of FER, which classifies faces into the corresponding expression categories based on the extracted features. Among the classical feature classification algorithms, support vector machines (SVM) [42] is by far the most widely used, followed by k-nearest neighbors (KNN) [43]. In addition, Adaboost [44], naive Bayes (NB) [45], and random forests (RF) [46] are also used for feature classification. However, as the complexity and diversity of facial expression data increases, these traditional methods are no longer applicable. As an end-to-end learning method, the neural network can output the probability of the category to which a sample belongs directly after feature extraction. The commonly used neural network-based classification algorithms are CNN [47] and multilayer perceptron (MLP) [39]. MLP is simpler and easier to study. However, the structure of fully connected layers will lose the spatial information between pixels. In contrast, CNN takes into account the spatial correlation of pixels and can achieve good results with fewer parameters. Therefore, most studies in recent years have used CNNs for feature classification.

## 3. Database Creation

From Section 2.1, we learned that most of the existing Asian facial expression datasets are collected in laboratory conditions. Furthermore, there are mainly three countries involved, namely, China, Japan, and Korea. To promote the development of Asian facial expression analysis in natural scenes, we decided to create a spontaneous East Asian facial expression database in natural scenes, which is dominated by China, Japan, and Korea. On one hand, spontaneous expressions can be compared with the posed expressions in existing lab-controlled datasets. We did this for two reasons. On the other hand, there is a great similarity in the facial appearances of these three countries due to the influence of geographical and ethnographic origins, so they can be fused to study.

### 3.1. Data Collection

Collecting various spontaneous expressions produced by people in real life is an extremely difficult task [48]. We cannot and do not have the conditions to monitor people’s behavior all the time. Movies are a form that reflects real social life through continuous video images, and they record people’s behavioral expressions in various natural scenes. Although movies are also shot in specific environments, they try to simulate the real world as much as possible to create an immersive feeling. Thus, the environment in the movie is closer to real scenes than the strictly controlled environments in laboratories [48]. It should be noted that although the expressions appearing in the movie are also performed by the actors, they are professionally trained to know how to control their expressions to imitate human behavior in real scenes [48]. Besides, the existing Asian facial expression database AFE and the widely used European-American facial expression database AFEW also collected images from movies. Based on the above analysis, we decided to collect expression data from movies.

**Figure 1:**
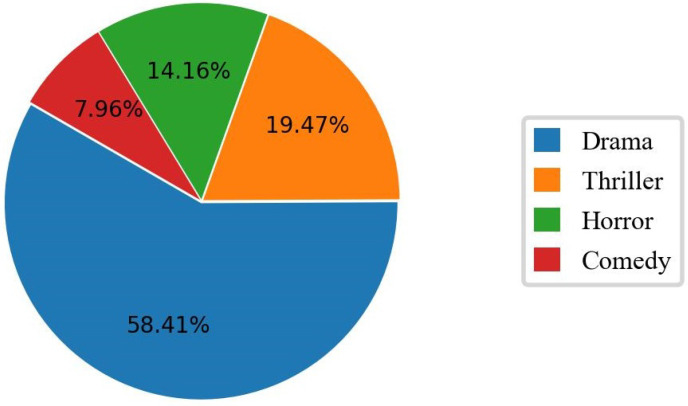
The proportion of different movie types among 113 movies.

However, the imaging was relatively blurry due to the limitation of shooting equipment in early movies, which is not conducive to extracting features. Therefore, we mainly collected high-rated movies from China, Japan, and Korea in the past 30 years. Because different types of movies focus on different emotions, the movie type was also taken into account to obtain a more balanced expression data. Finally, a total of 113 movies were selected, including 51 Chinese movies, 29 Japanese movies, and 33 Korean movies. All movie titles are presented in Appendix A. Figure 1 shows the proportion of different movie types, of which drama accounts for the largest proportion, reaching 58.4%, followed by thriller and horror. The length of movies ranged from 72 to 175 min, with an average length of 114 min.

Due to the design of the plot, it is hard to capture expressions such as disgust, fear, and surprise from movies. To alleviate the imbalance, we adapt the data collection method of AffectNet [3] (a large European-American facial expression database). We first defined a series of synonyms related to disgust, fear, and surprise, then combined them with attribute words such as age and gender to obtain phrases such as “surprised Chinese girl in the movie”. All expression synonyms and attribute words used are given in Table 2, making up a total of 162 phrases. We searched images in five search engines (Google, Bing, Baidu, Goo, and NAVER) based on these combined phrases. Specifically, we used Baidu, Goo, and NAVER to search for Chinese, Japanese, and Korean facial expression images, respectively. Then, we used Google and Bing to search for facial expression images from each of these three countries. However, as the images returned by the search engines were sorted according to their relevance to the search phrases, the later the images were, the less relevant they were. So, we only downloaded the first 100 images of the returned results each time. In the end, we obtained around 50,000 images from the web.

### 3.2. Data Processing

**Figure 2:**
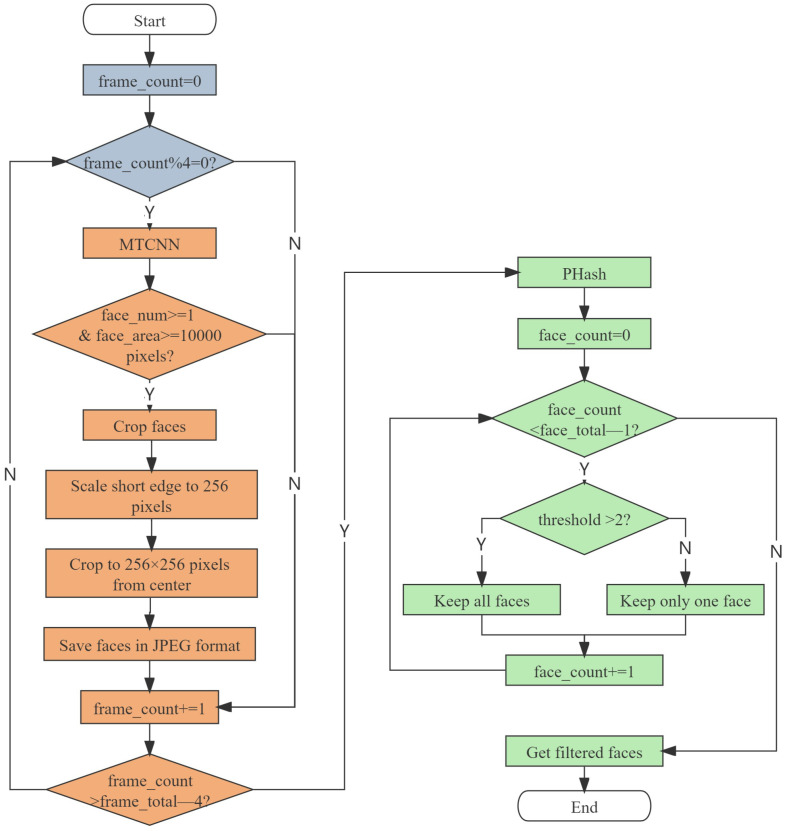
The automatic processing pipeline for filtering out faces from movies.

A continuous scene in a movie is composed of the basic frames, usually 24 frames per second, for a total of 18,550,080 frames in the selected 113 movies. Selecting facial expressions directly from these frames is an extremely challenging and time-consuming task. Therefore, we designed an automatic processing pipeline to filter out useful faces in movies. The filtering process is shown in Figure 2, where the blue parts represent “frame extraction”, the orange parts represent “face detection”, and the green parts represent “image deduplication”.

Frame extraction. For a movie, one second is a very short time. Unless subjected to a very large external stimulus, there will usually not be a particularly large change in action. There are therefore many repetitions in 24 frames, which do not provide additional information. To improve the speed of data processing, we adopted video frame extraction, which extracts one frame at a certain frame interval. Considering that the larger the interval, the more likely it is to lose instant expressions, such as surprise, we finally chose to extract one image from every four frames for further processing.

Face detection. Some frames used to create the atmosphere in the movies contain no human face, and they need to be eliminated. Furthermore, for frames with faces, only facial parts need to be preserved. As described in Section 2.2, the MTCNN algorithm achieves more robust face detection results in complex scenes. Considering the variety of head poses and illuminations in movies, we chose the robust MTCNN algorithm to perform face detection. If the frame contained no face, it was discarded directly. If detected to contain at least one face and the face area was larger than 10,000 pixels (to ensure face clarity), the short edge of the face was scaled to 256 pixels while keeping the aspect ratio unchanged, and then the face was cropped to 256 × 256 pixels from the center and saved in JPEG format.

Image deduplication. The faces obtained after the previous two steps still have great duplication or similarity, which will increase the time cost of labeling. Therefore, we were required to further process the iamges to remove the duplicates. Hash algorithms are often used to calculate the similarity between images, including the average hash (AHash) algorithm [49], perceptual hash (PHash) algorithm [50], and difference hash (DHash) algorithm [51]. AHash is simple and fast, but it is easily affected by the mean value, resulting in low accuracy. PHash is a more robust hash algorithm with higher accuracy but less speed. For DHash, its speed and accuracy is between those of AHash and PHash. The amount of data is critical, and we do not want to lose any usable images. Therefore, we chose the high precision PHash algorithm to deduplicate the images. This algorithm can convert each images into 64-bit binary code and judge their similarity by calculating the Hamming distance between the binary codes of two images. For the same individual, there is perhaps only a slight difference between the different expressions, so we chose a smaller threshold of two. Those below the threshold were considered to be more similar, where only one of them is kept and the other will be rejected. After all the processing, we finally obtained about 400,000 images.

Images downloaded from the web may contain some cartoon characters, and different search engines may return the same results, so we used the same “face detection” and “image deduplication” methods to discard these images. After processing, we obtained about 20,000 images that meet the requirements.

### 3.3. Data Annotation

Considering that most of the studies are based on discrete expressions, we labeled the collected data with seven expressions (six basic expressions and neutral). We recruited three members from our lab as annotators. Each of them receives one hour of uniform training before annotation, including AUs defined in FACS and AU combinations of six basic discrete expressions. However, the real world is more complex and contains more AUs and their combinations [5], so this was just a basic reference to help annotators understand different expressions more quickly. After training, the three annotators were separated to judge 210 pre-selected images (30 images for each expression and all images were randomly shuffled to ensure that they did not affect the annotators). They were required to choose one of the seven basic categories to annotate. After the annotation was completed, the administrator reviewed it with the three annotators to ensure that they have fully understood how the expression categories are divided.

Because all images are obtained through automatic pre-processing, we cannot guarantee that they will all meet the requirements. For example, they may contain images that are not real faces or expressions that do not belong to the seven expressions (such as talking, sleepy, and bored). For images that did not meet the requirements, they were discarded during the annotation process. It should be noted that most of the images collected from the web were happy and neutral faces, because people tend to express positive expressions in public. Because we aimed to reduce the imbalance between different expressions, the images denoted as happy and neutral from the web were also discarded during annotation. For images that met the requirements, the three annotators may also have had different opinions. To ensure the validity and reliability of the annotations, only images with the same opinion of the three annotations were left, and the rest were removed. Finally, all images will be checked again by the administrator. If the administrator disagreed with the annotation, the images were also removed. In total, each image was agreed upon by four annotators to ensure the quality of all annotations.

After annotation, we obtained 8449 images from movies. The number of images corresponding to each expression is shown in the second row of Table 3. Most of them are happy, neutral, angry, and sad, which are common expressions in all types of movies. We obtained 1551 images from the web, as shown in the third row of Table 3. Although we used the keyword “in movie” during our search, the images returned from the web may also have come from TV series, reality variety shows, or real life, but the expressions are all spontaneous. Other than that, there is no difference between the images collected from movies and the web. Combining the labeled images from movies and the web, we obtained the East Asian Facial Expression (EAFE) database. The last row of Table 3 gives the number of each expression in EAFE. The neutral expression has 2578 images, while the disgust and the fear expressions only have 478 and 400 images, respectively. After expansion from the web, there is still an imbalance between the data, which is a direct result of the essence of expressions in the real world [3].

Compared with other existing Asian facial expression databases, EAFE collects expressions that are spontaneously generated in real scenes, so the data are more realistic and more practical than the expressions obtained under single laboratory conditions. Compared to the large real-world AFE database, EAFE has three advantages. First of all, the 54,601 images of the AFE represent the facial expressions of all 48 countries in Asia, while the 10,000 images of EAFE only represent the facial expressions of three East Asian countries, so the latter has more facial expressions corresponding to a single country. Second, AFE integrates the facial expressions of all Asian countries, which is relatively extensive. However, EAFE is aimed at the three East Asian countries (China, Japan, and Korea) which have the closest facial appearance, so it is a more refined study. Third, the largest class in AFE (happy) has 26,453 images, while the smallest class (fear) has only 1755 images and the gap ratio is 15. For EAFE, the largest class (neutral) has 2578 images, and the smallest class (fear) has 400 images, so the gap ratio is 6. Therefore, the class imbalance of EAFE is much lighter than that of AFE.

We want to obtain the most value out of the EAFE database, so in addition to annotating basic expressions, we intend to provide additional facial attribute information. As far as we know, there are only two European-American databases that provide facial attributes, AffectNet and RAF-DB. The former uses Microsoft Cognitive Face API and the latter uses Face++ API. However, Face++ API requires a fee to use and both provide similar attribute information, so we prefer to use Microsoft Cognitive Face API to extract facial attributes. The Microsoft Cognitive Face API is provided by Microsoft Azure. It uses AI algorithms to detect, recognize, and analyze faces in images with high accuracy. In the face detection stage, a set of face-related attributes, such as age, gender, and head pose, can also be extracted. The detailed attributes and their values are shown in Table 4.

According to the Microsoft Cognitive Face API extraction results, 52% of the faces in EAFE are male and 48% are female. The ages range from 1 to 89, and the average age is 28.93 with a standard deviation of 9.8. As for the head pose, the average angle of yaw, pitch, and roll are 0.92, −12.49, and −2.3. The occlusion rates of the eye and mouth areas are 35.64% and 18.99%, respectively. Besides that, about 6.74% of people wear glasses. We saved all attribute results in the annotation documents in XML format so that future users can choose the appropriate images according to the attribute information to meet their needs.

## 4. Experiments and Results

In this section, we describe a series of experiments used to demonstrate the value of our database. Existing research on expression recognition shows that traditional handcrafted feature extraction methods are not suitable for complex scenes [3,5], while deep learning has definitive advantages due to its powerful ability to extract features. Therefore, we chose to use deep learning methods to carry out relevant experiments.

### 4.1. Pre-Processing

Before training the model, we divided all data into the training set, validation set, and testing set. The training set was used to train the model, the validation set was used to determine the best parameters, and the testing set was used to test the final performance of the model. To make the three sets have the same distribution, we first randomly shuffled all data. Then, we divided them by stratified sampling at a ratio of 8:1:1. Finally, we obtained 8000 training sets, 1000 validation sets, and 1000 testing sets.

We used data augmentation to increase the diversity of existing data. However, the training set was augmented differently than the validation and testing sets. During training, we used the common data augmentation strategy [52], including random resized crop and random horizontal flip, while for the validation and testing sets, we used a single-center crop, and no other random augmentation methods were used.

### 4.2. Training Procedures

We chose three CNNs commonly used in FER: VGG-16 [53], ResNet-50 [54], and Inception-V3 [55] as different network architectures. To explore the effect of different types of learning rate schedules and loss functions on model performance, we conducted two sets of comparative experiments. During the experiments, all networks were initialized with pretrained weights using ImageNet [56], then trained on a single Tesla V100. The detailed configuration is shown in Table 5. Because there was a lot of randomness in the training process, we used a fixed seed to make the results comparable and reproducible.

#### 4.2.1. Learning Rate Schedule

The learning rate is perhaps the most important hyperparameter in networks [57], which controls the rate of weight updates during training. In general, we hope to have a larger learning rate at the beginning of training to make the network converge quickly and a smaller learning rate at the end of training to make the network converge better to the optimal solution. The way in which the learning rate changes over time is called the learning rate schedule (LR schedule). Our first set of experiments focused on finding an optimal learning rate schedule.

**Figure 3:**
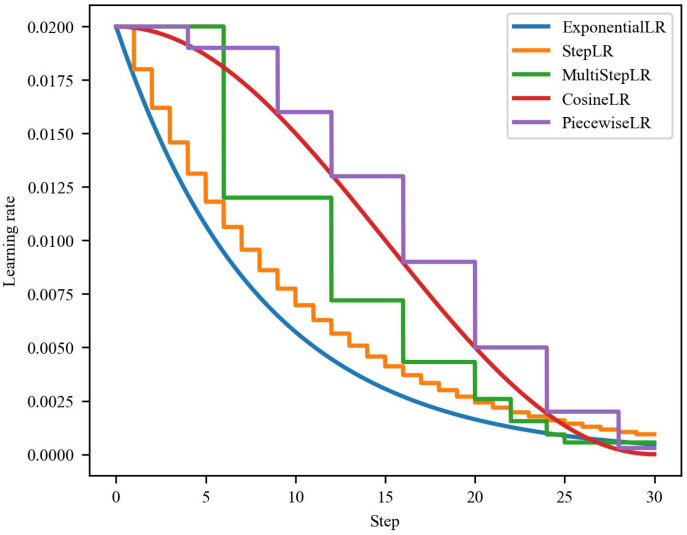
The learning rate decay process of five learning rate schedules.

We selected five commonly used learning rate schedules, including the exponential LR schedule (ExponentialLR) [58], which decays the learning rate exponentially by multiplying the same factor every epoch; the step decay LR schedule (StepLR) [59], that decays the learning rate in the same proportion after a fixed step; the multistep decay LR schedule (MultiStepLR), which decays the learning rate in the same proportion after different iterations; the cosine LR schedule (CosineLR) [60], which decays the learning rate as a cosine curve and the update is slow at the beginning; and piecewise constant decay LR schedule (PiecewiseLR) which decays the learning rate by setting different learning rates after different iterations. Figure 3 shows the learning rate decay process using these learning rate schedules. We can see that they sweep most of the possible decay values. Although StepLR, MultiStepLR, and PiecewiseLR are similar in decreasing the learning rate after a certain step, their update methods are different, resulting in different weight updates.

A grid search was used to determine the best initial learning rate for each network and the parameters for each learning rate schedule. For each network, once the optimal initial learning rate is found, all learning rate schedules will start decaying. During the experiment, we trained all networks optimizing cross-entropy loss and kept the other parameters fixed.

#### 4.2.2. Loss Function

The purpose of the second set of experiments was to find the most appropriate loss function. Besides cross-entropy loss, center loss [61], and island loss [62] are two loss functions commonly used in FER research. Center loss provides the class center for each class and then narrows the intra-class gap by minimizing the distance between samples and class centers. It is defined as:

(1) $$L_{C}=\frac{1}{2}\sum_{i=1}^{m}{∥x_{i}-c_{y_{i}}∥}_{2}^{2}$$

where *m* denotes the mini-batch size, $x_{i}$ denotes the features of *i*th sample, $c_{y_{i}}$ denotes the feature center of the $y_{i}$th class.

Island loss combines the idea of center loss to reduce the intra-class gap while expanding the inter-class gap so that the network can learn more discriminative features. It is defined as:

(2) $$L_{IL}=L_{C}+\lambda_{1}\sum_{c_{j}\in N}\sum_{\begin{array}{c} c_{k}\in N\\ c_{k}\neq c_{j} \end{array}}\left(\frac{c_{k}\cdot c_{j}}{{∥c_{k}∥}_{2}{∥c_{j}∥}_{2}}+1\right)$$

where $L_{C}$ denotes the center loss, N denotes the set of expression labels, $c_{k}$ and $c_{j}$ denote the *k*th and *j*th center with $L_{2}$ norm ${∥c_{k}∥}_{2}$, and ${∥c_{j}∥}_{2}$, $\lambda_{1}$ denotes the balance coefficient.

The parameters involved in these loss functions were determined using a grid search. We used the optimal learning rate schedule determined by the first set of experiments and kept the other parameters fixed during this experiment.

### 4.3. Experimental Results

The distribution of facial expression data in EAFE is unbalanced, which is very sensitive to bias [63]. For the comparability of results, we used th e average accuracy as the evaluation metric. It averages the accuracy for each class and pays more attention to classes with fewer samples.

#### 4.3.1. Results on LR Schedule

**Figure 4:**
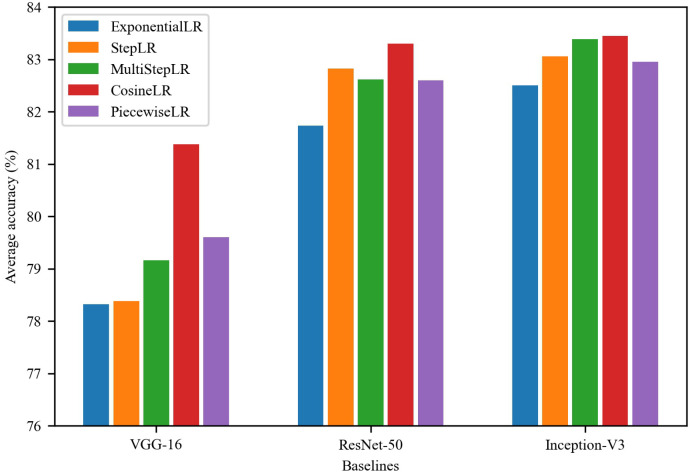
Experimental results of different LR Schedules on the validation set.

Figure 4 shows the evaluation results of different learning rate schedules on the validation set. In all experimental results, CosineLR outperforms the other learning rate schedules, especially for VGG-16. It is mainly because of the slow decay in the later stages, which allows the network to converge better. The performances of StepLR, MultiStepLR, and PiecewiseLR are comparable. Among them, StepLR is better than the other two in ResNet-50, while MultiStepLR shows an advantage in Inception-V3, reaching an accuracy close to that of CosineLR. Unlike the previous two, PiecewiseLR is more suitable for VGG-16. ExponentialLR performs the worst among all learning rate schedules, considering that the initial learning rate decays too fast, resulting in the model cannot converge well. Comparing the performance of different neural networks, Inception-V3 performs the best, followed by ResNet-50, and the worst is VGG-16, which is positively correlated with their model parameters.

#### 4.3.2. Results on Loss Function

**Figure 5:**
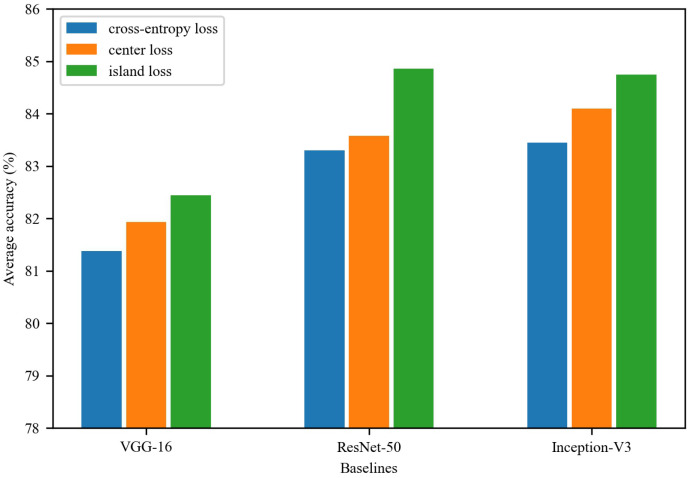
Experimental results of different loss functions on the validation set.

In the second set of experiments, we use the best learning rate schedule based on the previous section, CosineLR. Figure 5 shows the evaluation results of different loss functions on the validation set. In all experiments, island loss performs best, especially for ResNet-50, achieving an accuracy of 84.86% in the validation set. It is mainly because island loss considers both the intra-class distance and the inter-class distance at the same time. Center loss, on the other hand, performs second best because only intra-class distance is considered. The worst is cross-entropy loss, but it also achieves an accuracy of 81.38% on VGG-16.

#### 4.3.3. Results on Testing Set

According to the previous analysis, all three CNNs can achieve the best results on the validation set when using CosineLR and island loss. Therefore, we use the models trained based on the two methods to do the final test on the testing set. Table 6 shows the total results. ResNet-50 has the best overall recognition result with an accuracy of 80.53%, followed by Inception-V3 and VGG-16. As for each expression, happy and surprise are easier to recognize, but disgust and fear have poor recognition results. The main reason for this is that the latter has less training data compared to the former. This unbalanced distribution will lead to the models not being able to fully learn the features of fewer categories, resulting in poor generalization ability [3].

**Figure 6:**
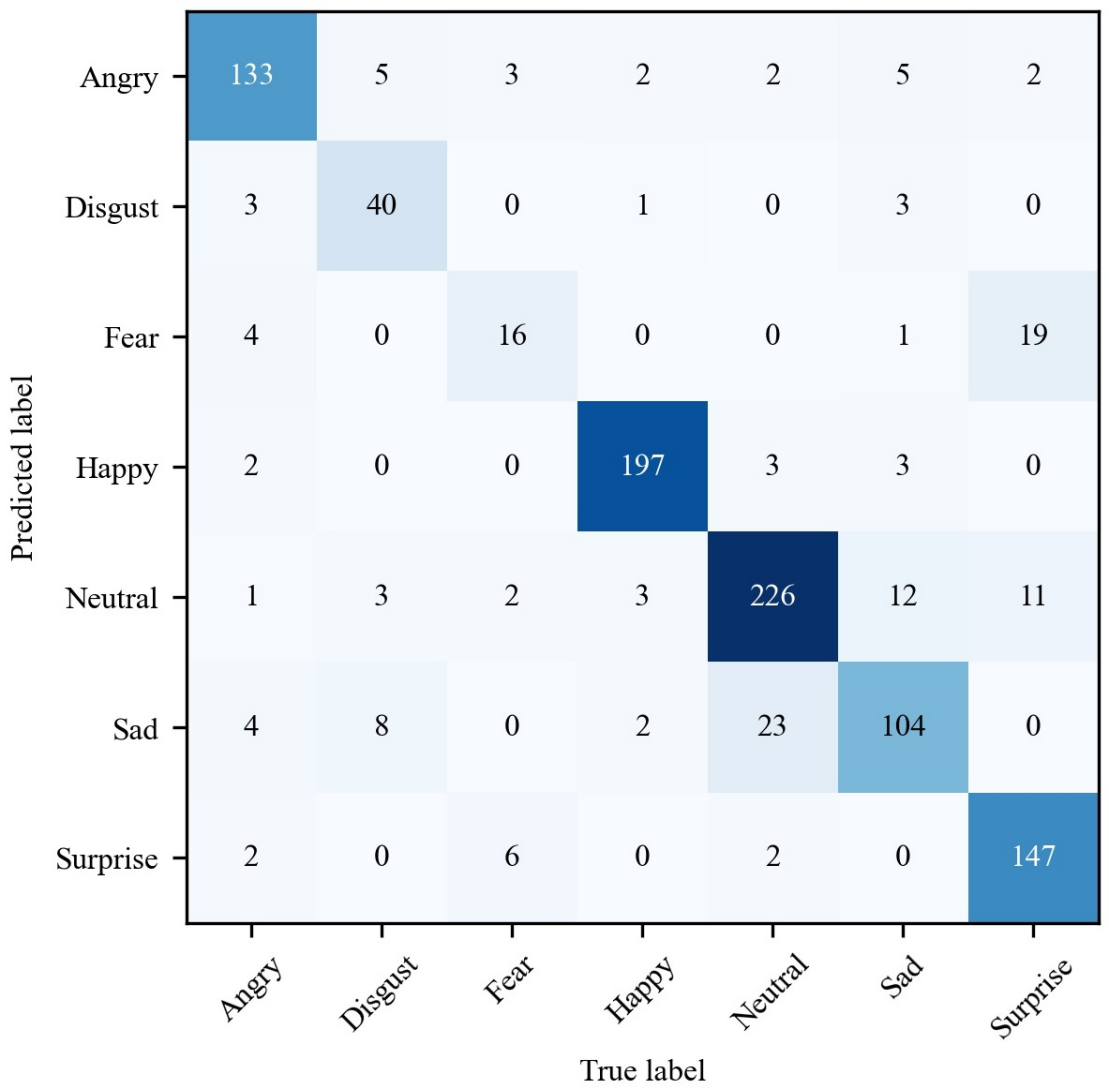
Confusion matrix of the baseline ResNet-50 evaluated on the testing set.

Figure 6 shows the confusion matrix of ResNet-50 on the testing set, where each row represents the true labels and each column represents the predicted labels. The numbers on the diagonal line represent the correctly classified expressions while the non-diagonal numbers represent the misclassified expressions. We can see that most samples can be classified correctly. Fear is most likely to be confused with surprise, with about 47.5% being misclassified. This phenomenon is similar to other Asian facial expression recognition research [20]. It may be due to the great similarity of the two expressions, such as raised eyebrows, widened eyes, and open mouth. In addition, sad and neutral expressions are also very easy to be confused. The main reason is that some sad expressions are subtle, such as melancholy which can only be seen from the eyes. However, affected by various head poses, it is difficult to capture this kind of performance characteristics, making it difficult to distinguish sad from neutral. All results demonstrate that expressions in real scenes are not as obvious and easily distinguishable as those posed in laboratory conditions. In addition, due to the uncontrolled spontaneity, the same expressions are expressed in a variety of ways, which increases the difficulty of recognition.

## 5. Conclusions

In this paper, we designed an automatic processing pipeline and used it to capture about 450,000 images from 113 Chinese, Japanese, and Korean movies and five search engines (Google, Bing, Baidu, Goo, and NAVER). Three trained annotators annotated these images based on seven discrete expressions (angry, disgust, fear, happy, neutral, sad, and surprise). Only images where all annotations were the same were retained. In the end, 10,000 expression images were obtained, making up the real-world EAFE database. In addition to the seven expressions, we used the Microsoft Cognitive Face API to extract facial attributes such as age, gender, and head rotation angle. Thus, the database can be used not only for facial expression analysis but also for facial recognition and attribute analysis. Three neural network baselines were used to classify the facial expression images in EAFE. We then explored the effects of different learning rate schedules and loss functions on these networks. Finally, by training with the cosine learning rate schedule and island loss, ResNet-50 achieved a best accuracy of 80.53% on the testing set, proving that the database is challenging. We hope that the release of the EAFE will encourage more research on Asian FER, and also promote the development of FER in cross-cultural domains.

## Figures and Tables

**Table 1:** Details of the existing Asian facial expression databases.

Database	Year	Ethnicity	Co. ^1^	El. ^2^	No. of Images	Su. ^3^	Resolution	Expressions
JAFFE	1998	Japanese	Lab	P ^4^	213	10	256 × 256	six basic expressions, neutral
TFEID	2007	Chinese	Lab	P	7200	40	600 × 480	six basic expressions, contempt, neutral
PF07	2008	Korean	Lab	P	64,000	200	640 × 480	angry, happy, neutral, surprise
NVIE	2010	Chinese	Lab	P	–	108	704 × 480	six basic expressions
				S ^5^		215		
CAFPS	2011	Chinese	Lab	P	870	220	260 × 300	six basic expressions, neutral
KTFE	2013	V-T-J ^6^	Lab	S	330	26	–	six basic expressions, neutral
KUFEC-II	2017	Korean	Lab	P	399	57	1280 × 1024	six basic expressions, neutral
TFEES	2018	Chinese	Lab	P	3709	90	2592 × 3872	six basic expressions, neutral
KRC	2019	Japanese	Lab	P	4736	74	851 × 933	six basic expressions, neutral
Tsinghua FED	2020	Chinese	Lab	S	1128	110	1800 × 2200	six basic expressions, content, neutral
MMEW	2021	Chinese	Lab	S	300 (Micro)	30	231×231	six basic expressions
					900 (Macro)			
AFE	2021	Asian	Movie	S	54,601	–	112×112	six basic expressions, neutral

^1^ Collection condition. ^2^ Elicitation method. ^3^ Number of subjects. ^4^ Posed. ^5^ Spontaneous. ^6^ Vietnamese, Thai, Japanese.

**Table 2:** The expression synonyms and attribute words used in search engines.

Expression	Age and Gender	Nation
Disgusted	Girl	Chinese
Detestable	Boy	Japanese
Disliked	Man	Korean
Fearful	Woman	
Frightened	Old man	
Horrible	Old lady	
Surprised		
Amazed		
Astonished		

**Table 3:** The number of expressions collected from movies and the web.

Collection Condition	Angry	Disgust	Fear	Happy	Neutral	Sad	Surprise	Total
Movie	1417	78	207	2050	2578	1245	874	8449
Web	102	395	193	0	0	164	697	1551
Movie & Web	1519	473	400	2050	2578	1409	1571	10,000

**Table 4:** The detail attributes and values provided by the Microsoft Cognitive Face API.

Attribute	Value
Gender	male, female
Age	specific numbers
Head pose	yaw, pitch, roll (−180°–180°)
Occlusion	eyeOccluded, mouthOccluded (true or false)
Glasses	NoGlasses, ReadingGlasses, Sunglasses, Swimming Goggles

**Table 5:** The parameter configuration in all experiments.

Parameter	Value
Optimizer	stochastic gradient descent
Momentum	0.9
Weight decay	0.0001
Epoch	30
Batch size	64

**Table 6:** The recognition results of VGG-16, ResNet-50, and Inception-V3 on the testing set.

Network	Angry	Disgust	Fear	Happy	Neutral	Sad	Surprise	Average
VGG-16	86.18	70.21	35.00	91.71	84.50	75.18	85.35	75.45
ResNet-50	87.50	85.11	40.00	96.10	87.60	73.76	93.63	**80.53**
Inception-V3	88.16	74.47	40.00	92.68	86.82	78.01	88.54	78.38

## Data Availability

The data presented in this study are available on request from the corresponding author.

## Abbreviations

The following abbreviations are used in this manuscript:

FER	Facial Expression Recognition
JAFFE	Japanese Female Facial Expression
TFEID	Taiwanese Facial Expression Image Database
PF07	POSTECH Face Database
NVIE	Natural Visible and Infrared Facial Expression Database
CAFPS	Chinese Affective Face Picture System
KTFE	Kotani Thermal Facial Emotion
KUFEC-II	Korea University Facial Expression Collection-Second Edition
TFEES	Taiwanese Facial Emotional Expression Stimuli
KRC	Kokoro Research Center Facial Expression Database
Tsinghua FED	Tsinghua Facial Expression Database
MMEW	Micro-and-macro Expression Warehouse
AFE	Asian Face Expression
MTCNN	Multi-task Convolutional Neural Network
LBP	Local Binary Pattern
LDP	Local Derivative Pattern
WPLBP	Weighted-projection-based LBP
HOG	Histogram of Oriented Gradient
PHOG	Pyramid Histogram of Oriented Gradients
PCA	Principal Component Analysis
SWLDA	Stepwise Linear Discriminant Analysis
LCT	Local Curvelet Transform
CNN	Convolutional Neural Network
DBN	Deep Belief Network
DAE	Deep Autoencoder
GAN	Generative Adversarial Network
SVM	Support Vector Machines
KNN	K-Nearest Neighbors
NB	Naive Bayes
RF	Random Forest
MLP	Multilayer Perceptron
AU	Action Unit
FACS	Facial Action Coding System
AHash	Average Hash
PHash	Perceptual Hash
DHash	Difference Hash
EAFE	East Asian Facial Expression
LR	Learning Rate
ExponentialLR	Exponential LR Schedule
StepLR	Step Decay LR schedule
MultiStepLR	MultiStep Decay LR schedule
CosineLR	Cosine LR schedule
PiecewiseLR	Piecewise Constant Decay LR schedule

## Appendix A

Table A1 lists the details of 113 movies selected from the past 30 years, sorted by year and nation.

**Table A1:** Details of the 113 movies.

Year	Nation	Type	Title
1989	China	Horror	Ghost Busting
1993	China	Horror	Horror School
1993	China	Drama	Insanity
1995	China	Horror	01:00 a.m.
2000	China	Horror	Dial D for Demons
2002	China	Thriller	Inner Senses
2002	China	Horror	Ghost Office
2006	China	Drama	Little Red Flowers
2007	China	Drama	Protege
2010	China	Drama	Aftershock
2010	China	Horror	Curse of the Deserted
2010	China	Horror	Midnight Beating
2012	China	Drama	Tao Jie
2012	China	Drama	Cold War
2012	China	Horror	The Mask of Love
2013	China	Drama	American Dreams in China
2013	China	Drama	Drug War
2013	China	Horror	The House
2013	China	Horror	The Supernatural Events on Campus
2015	China	Drama	Our Times
2015	China	Comedy	Goodbye Mr. Loser
2015	China	Drama	The Dead End
2015	China	Drama	Blind Spot
2016	China	Drama	Soulmate
2016	China	Thriller	Lost in Apocalypse
2017	China	Thriller	Shock Wave
2017	China	Drama	Angels Wear White
2017	China	Thriller	Guilty of Mind
2017	China	Thriller	Always Be with You
2018	China	Drama	Dying to Survive
2018	China	Comedy	A Cool Fish
2018	China	Drama	Project Gutenberg
2018	China	Drama	Wrath Of Silence
2019	China	Drama	Better Days
2019	China	Drama	The Wandering Earth
2019	China	Drama	Me and My Motherland
2019	China	Comedy	Hello Mr. Billionaire
2019	China	Drama	So Long, My Son
2019	China	Comedy	Almost a Comedy
2019	China	Drama	A Sun
2019	China	Drama	Sheep Without a Shepherd
2019	China	Comedy	King of comedy
2019	China	Drama	The Captain
2020	China	Drama	A Little Red Flower
2020	China	Drama	Duo Guan
2020	China	Drama	My People My Homeland
2020	China	Drama	Little Big Women
2021	China	Comedy	Hi, Mom
2021	China	Drama	Be Somebody
2021	China	Drama	Break Through the Darkness
2021	China	Drama	Fireflies in the Sun
1996	Japan	Drama	Picnic
2005	Japan	Horror	Futago
2009	Japan	Drama	Elevator Trap
2012	Japan	Comedy	I AM a HERO
2013	Japan	Drama	Tokyo Kazoku
2013	Japan	Thriller	The Werewolf Game: The Villagers Side
2013	Japan	Drama	Like Father Like Son
2014	Japan	Horror	Bilocation
2014	Japan	Drama	Killers
2014	Japan	Thriller	The Werewolf Game: The Beast Side
2016	Japan	Horror	Parasyte
2016	Japan	Thriller	Creepy
2016	Japan	Thriller	Museum
2017	Japan	Drama	Miracles of the Namiya General Store
2017	Japan	Thriller	The Devotion of Suspect X
2017	Japan	Drama	Memoirs of a Murderer
2017	Japan	Drama	Birds Without Names
2017	Japan	Thriller	The Scythian Lamb
2017	Japan	Drama	Manhunt
2017	Japan	Thriller	Traces of Sin
2018	Japan	Drama	The Crimes That Bind
2018	Japan	Drama	Shoplifters
2018	Japan	Thriller	The Blood of Wolves
2018	Japan	Drama	Born Bone Born
2019	Japan	Thriller	The Masquerade Hotel
2019	Japan	Drama	Killing For the Prosecution
2019	Japan	Drama	Day and Night
2020	Japan	Drama	Future Family
2021	Japan	Drama	Wheel of Fortune and Fantasy
2004	Korea	Drama	When I Turned Nine
2006	Korea	Thriller	Apartment
2006	Korea	Horror	The Host
2007	Korea	Horror	Harpy
2008	Korea	Drama	The Chaser
2008	Korea	Comedy	Baby and Me
2009	Korea	Drama	Tsunami
2009	Korea	Thriller	Murder in Itaewon
2009	Korea	Thriller	Mother
2010	Korea	Thriller	The Man from Nowhere
2011	Korea	Thriller	Sector7
2012	Korea	Horror	The Neighbors
2012	Korea	Thriller	Knock
2012	Korea	Drama	Unbowed
2012	Korea	Drama	Pieta
2013	Korea	Drama	Miracle in Cell No.7
2013	Korea	Drama	Byeonhoin
2013	Korea	Drama	The Flu
2013	Korea	Drama	New World
2015	Korea	Drama	Inside Men
2016	Korea	Drama	Canola
2016	Korea	Thriller	Train to Busan
2016	Korea	Drama	Murder at Honeymoon Hotel
2016	Korea	Drama	The Tunnel
2016	Korea	Thriller	Tik Tok
2017	Korea	Drama	I Can Speak
2018	Korea	Comedy	EXIT
2019	Korea	Drama	Gisaengchung
2019	Korea	Drama	Kim Ji-young: Born 1982
2019	Korea	Drama	The Gangster
2020	Korea	Drama	The Call
2020	Korea	Drama	Alive
2021	Korea	Drama	Raging Bull
